# Supplementary material for: Circulating immune profiling reveals impaired monocyte states and trajectories driving immunosuppression in glioblastoma
Source: J Neuroinflammation. 2026 Jul 16;23:306. doi: 10.1186/s12974-026-03964-3 (PMC13555986; doi:10.1186/s12974-026-03964-3)
Supplement: Supplementary file 1 [file 12974_2026_3964_MOESM1_ESM.zip › Fig S10_AS.pdf]

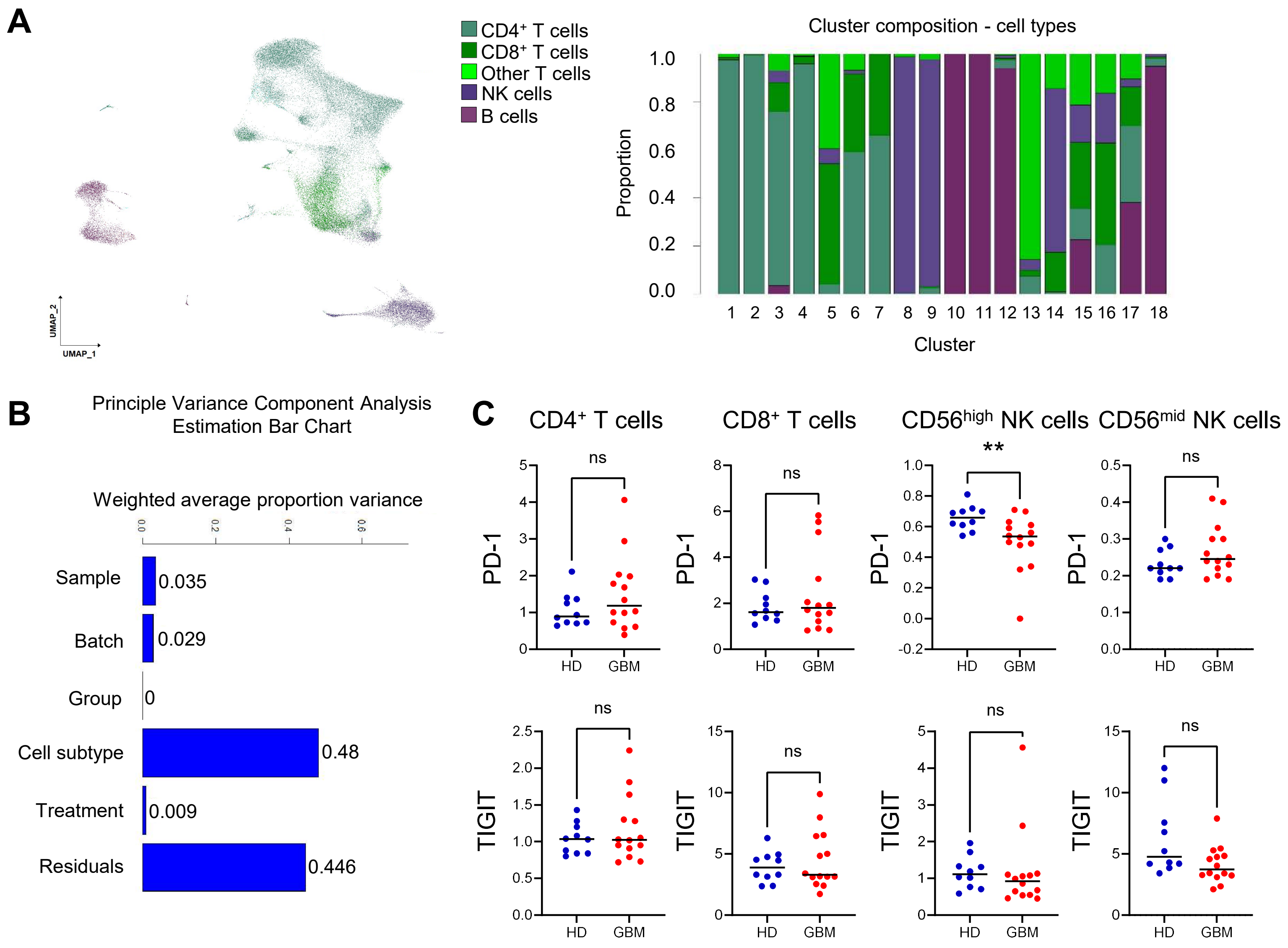
A
CD4+ T cells
CD8+ T cells
Other T cells
NK cells
B cells
UMAP_2
UMAP_1
Cluster composition - cell types
Proportion
Cluster
B
Principle Variance Component Analysis
Estimation Bar Chart
Weighted average proportion variance
Sample 0.035
Batch 0.029
Group 0
Cell subtype 0.48
Treatment 0.009
Residuals 0.446
C
CD4+ T cells
CD8+ T cells
CD56high NK cells
CD56mid NK cells
PD-1
TIGIT
ns
**
HD
GBM
