# Supplementary material for: Circulating immune profiling reveals impaired monocyte states and trajectories driving immunosuppression in glioblastoma
Source: J Neuroinflammation. 2026 Jul 16;23:306. doi: 10.1186/s12974-026-03964-3 (PMC13555986; doi:10.1186/s12974-026-03964-3)

A Interaction myeloid cell ligand - myeloid / lymphoid cell receptor

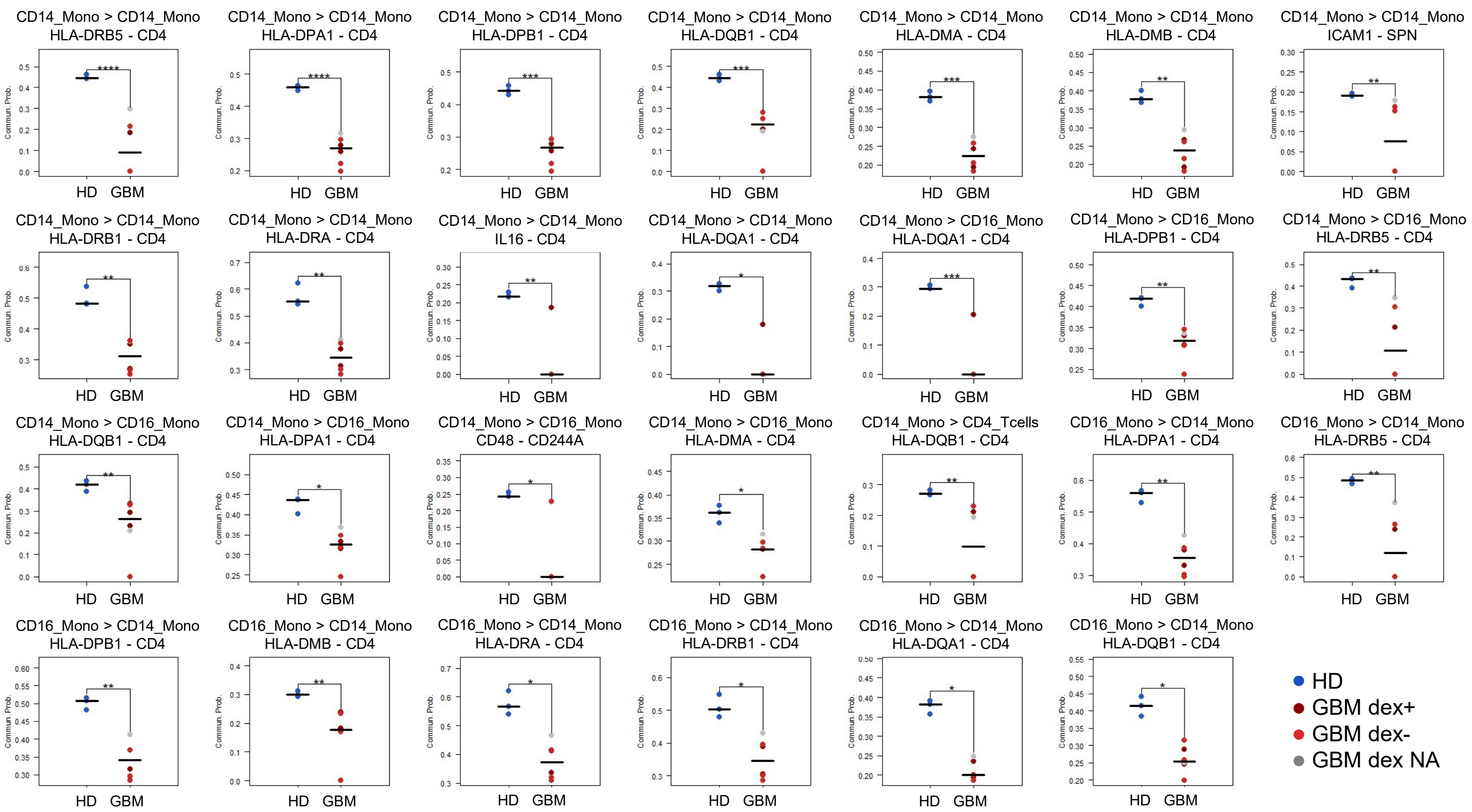

B Interaction lymphoid cell ligand - myeloid / lymphoid cell receptor

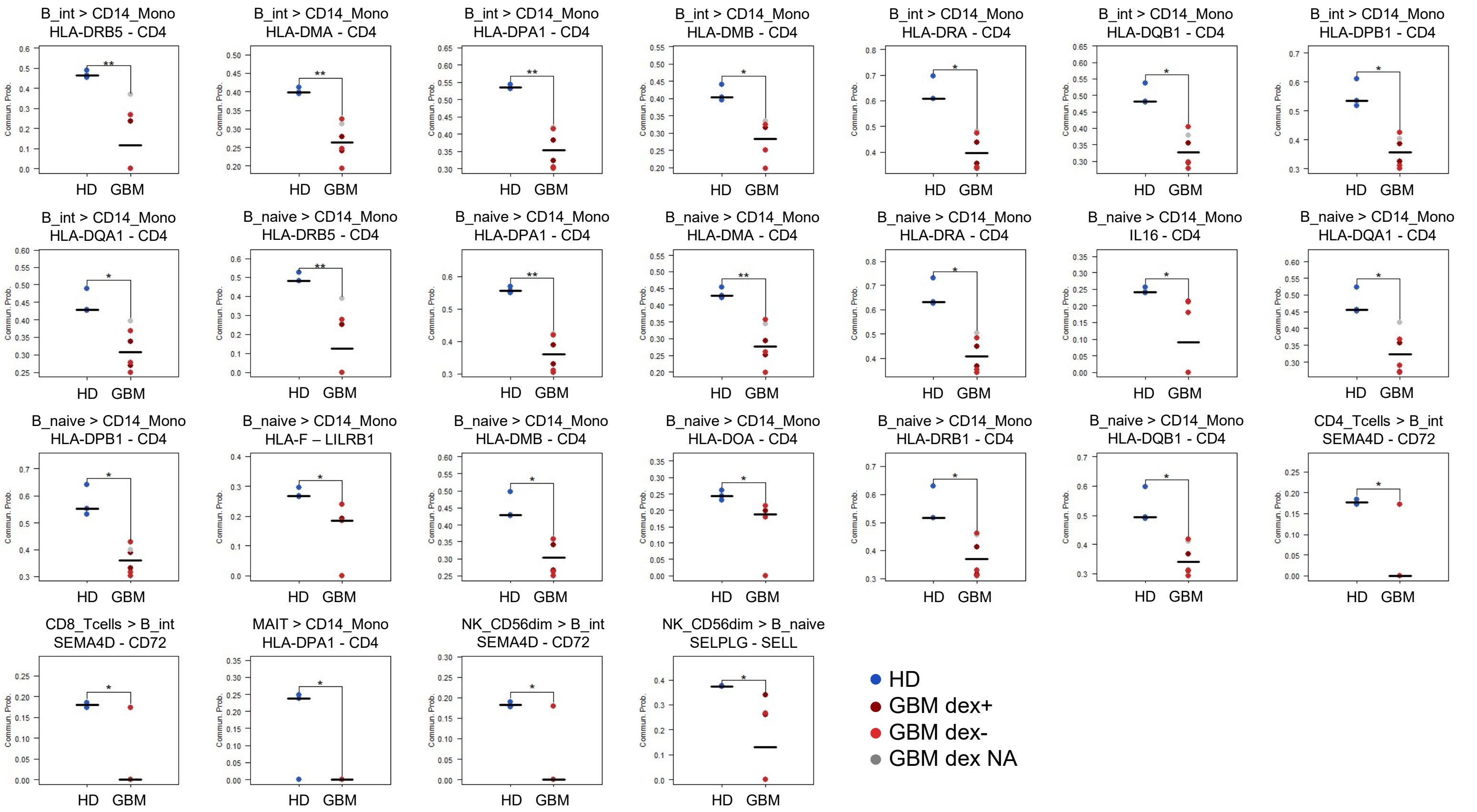

Supplement: Supplementary file 1 [file 12974_2026_3964_MOESM1_ESM.zip › Fig S11_AS.pdf]
