# Supplementary material for: Circulating immune profiling reveals impaired monocyte states and trajectories driving immunosuppression in glioblastoma
Source: J Neuroinflammation. 2026 Jul 16;23:306. doi: 10.1186/s12974-026-03964-3 (PMC13555986; doi:10.1186/s12974-026-03964-3)
Supplement: Supplementary file 1 [file 12974_2026_3964_MOESM1_ESM.zip › Fig S1_AS.pdf]

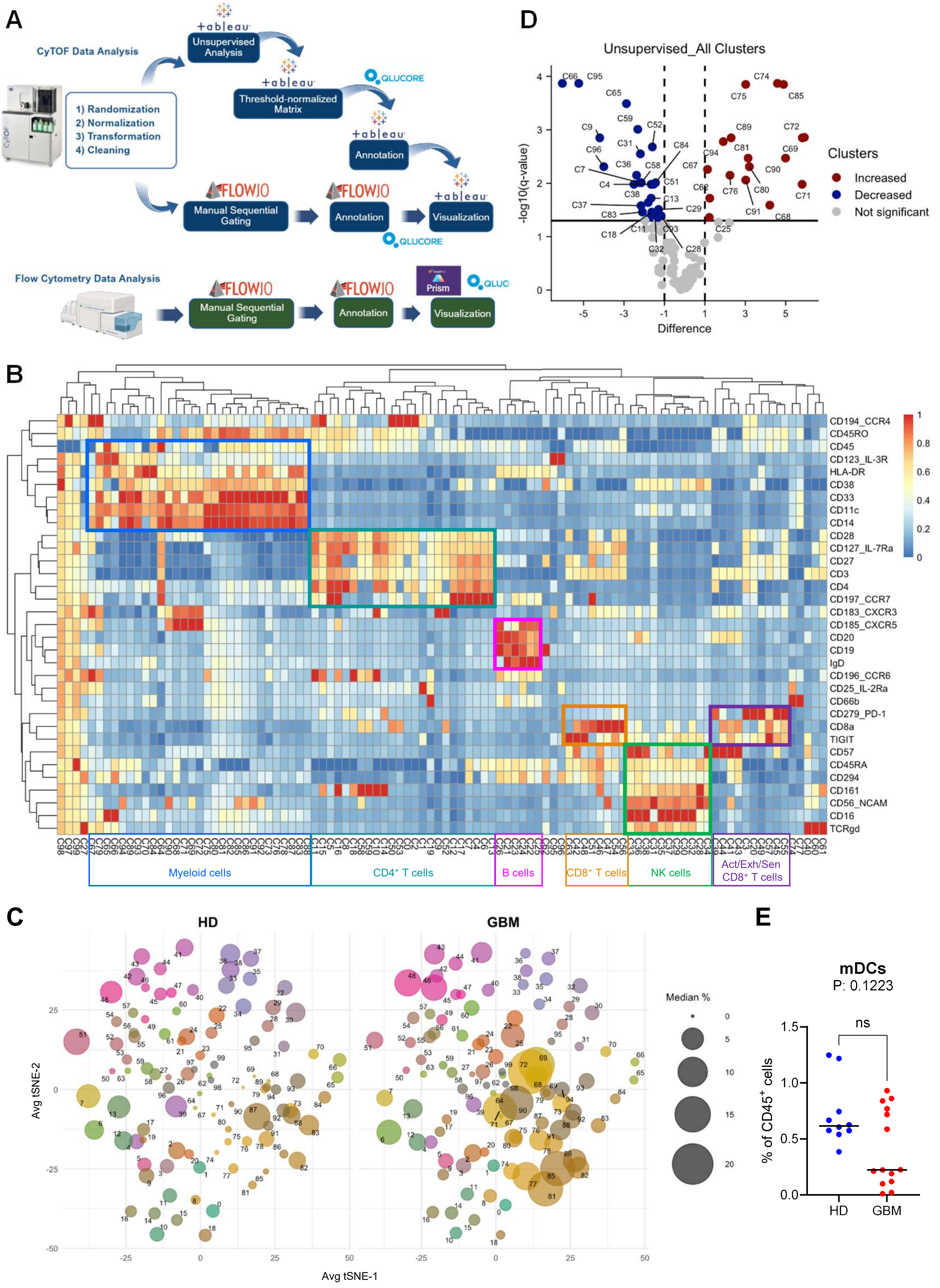
A
CyTOF Data Analysis
1) Randomization
2) Normalization
3) Transformation
4) Cleaning
Unsupervised Analysis
Threshold-normalized Matrix
Annotation
Manual Sequential Gating
Annotation
Visualization
Flow Cytometry Data Analysis
Manual Sequential Gating
Annotation
Prism
Visualization
D
Unsupervised_All Clusters
-log10(q-value)
Difference
Clusters
Increased
Decreased
Not significant
B
CD194_CCR4
CD45RO
CD45
CD123_IL-3R
HLA-DR
CD38
CD33
CD11c
CD14
CD28
CD127_IL-7Ra
CD27
CD3
CD4
CD197_CCR7
CD183_CXCR3
CD185_CXCR5
CD20
CD19
IgD
CD196_CCR6
CD25_IL-2Ra
CD66b
CD279_PD-1
CD8a
TIGIT
CD57
CD45RA
CD294
CD161
CD56_NCAM
CD16
TCRgd
Myeloid cells
CD4⁺ T cells
B cells
CD8⁺ T cells
NK cells
Act/Exh/Sen CD8⁺ T cells
C
HD
GBM
Avg tSNE-2
Avg tSNE-1
Median %
E
mDCs
P: 0.1223
ns
% of CD45⁺ cells
HD
GBM
