# Supplementary material for: Circulating immune profiling reveals impaired monocyte states and trajectories driving immunosuppression in glioblastoma
Source: J Neuroinflammation. 2026 Jul 16;23:306. doi: 10.1186/s12974-026-03964-3 (PMC13555986; doi:10.1186/s12974-026-03964-3)
Supplement: Supplementary file 1 [file 12974_2026_3964_MOESM1_ESM.zip › Fig S2_AS.pdf]

A

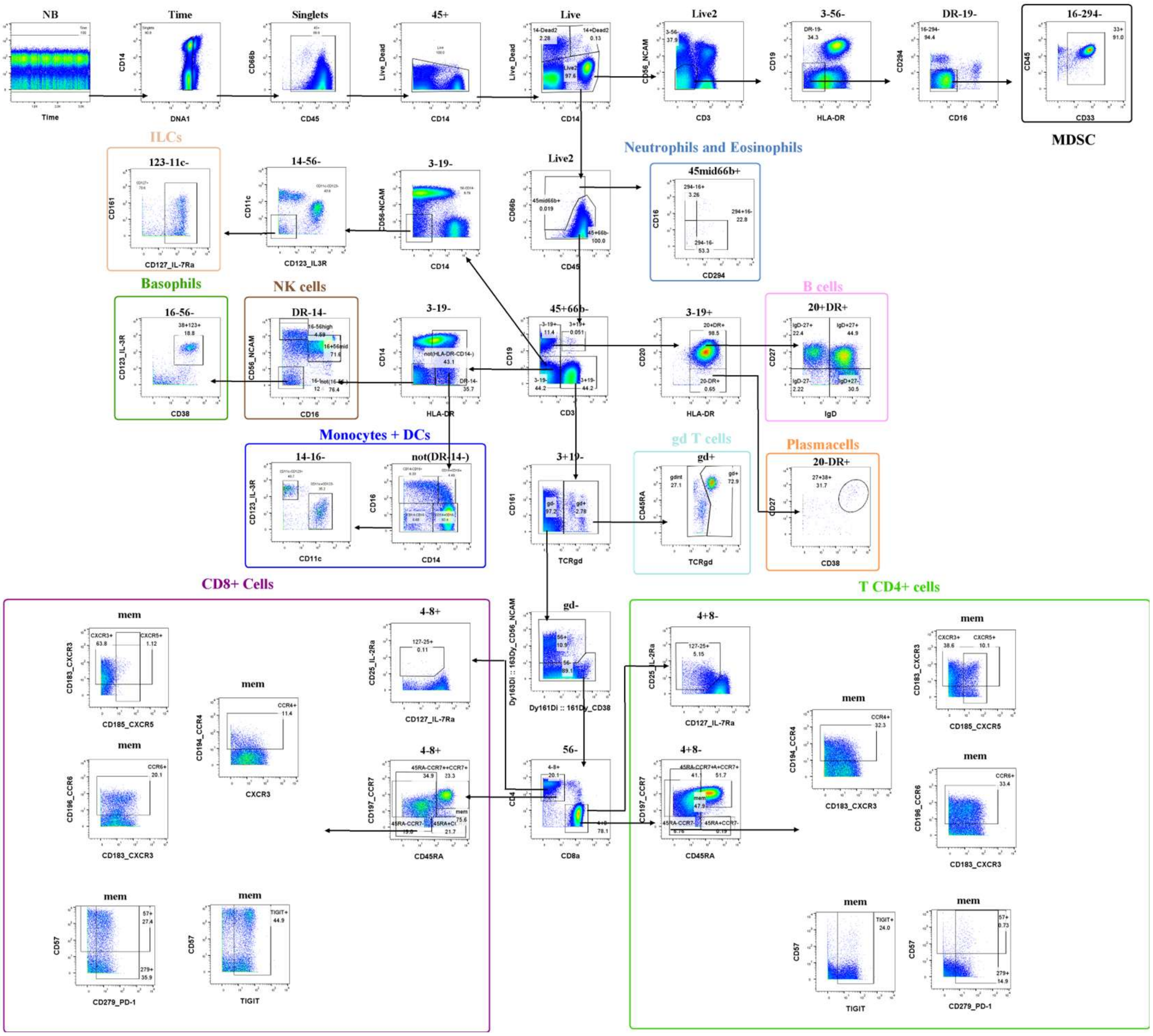

NB
Time
Singlets
45+
Live
Live2
3-56-
DR-19-
16-294-
MDSC
ILCs
123-11c-
14-56-
3-19-
Neutrophils and Eosinophils
45mid66b+
Basophils
16-56-
NK cells
DR-14-
45+66b-
3-19+
B cells
20+DR+
Monocytes + DCs
14-16-
not(DR-14-)
3+19-
gd T cells
gd+
Plasmacells
20-DR+
CD8+ Cells
T CD4+ cells
mem
4-8+
gd-
56-
4+8-

B

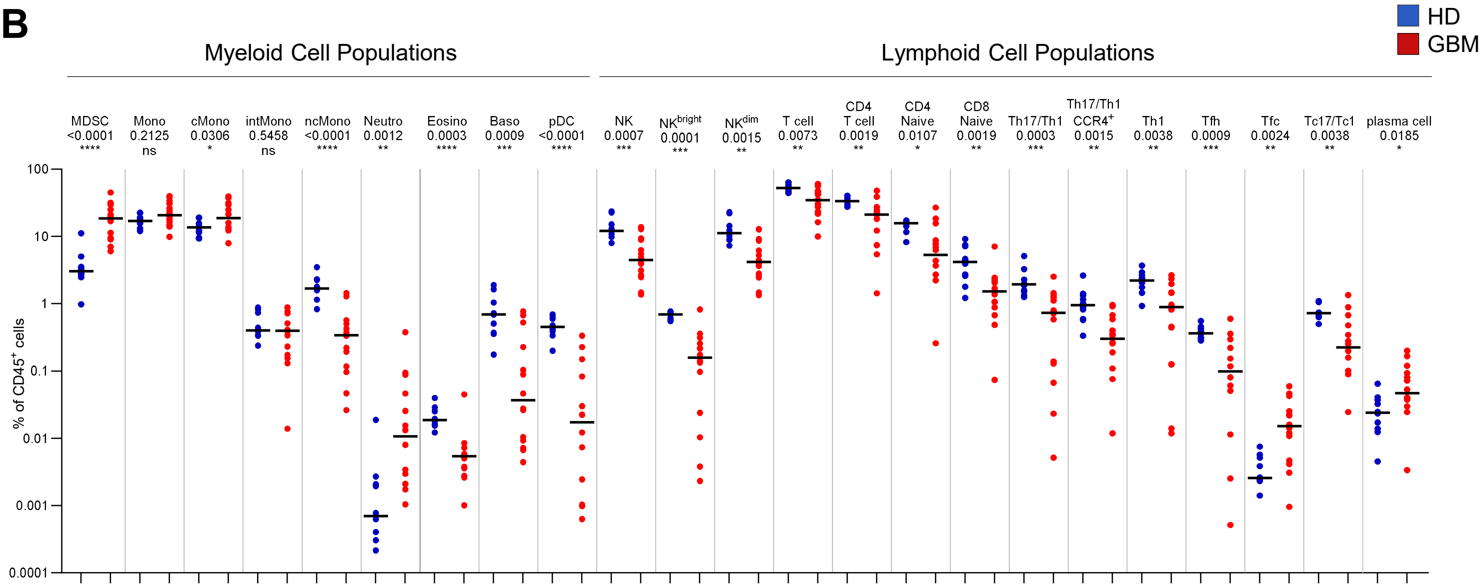

HD
GBM
Myeloid Cell Populations
Lymphoid Cell Populations
MDSC <0.0001 ****
Mono 0.2125 ns
cMono 0.0306 *
intMono 0.5458 ns
ncMono <0.0001 ****
Neutro 0.0012 **
Eosino 0.0003 ****
Baso 0.0009 ***
pDC <0.0001 ****
NK 0.0007 ***
NKbright 0.0001 ***
NKdim 0.0015 **
T cell 0.0073 **
CD4 T cell 0.0019 **
CD4 Naive 0.0107 *
CD8 Naive 0.0019 **
Th17/Th1 0.0003 ***
Th17/Th1 CCR4+ 0.0015 **
Th1 0.0038 **
Tfh 0.0009 ***
Tfc 0.0024 **
Tc17/Tc1 0.0038 **
plasma cell 0.0185 *
% of CD45+ cells
100
10
1
0.1
0.01
0.001
0.0001
