# Supplementary material for: Circulating immune profiling reveals impaired monocyte states and trajectories driving immunosuppression in glioblastoma
Source: J Neuroinflammation. 2026 Jul 16;23:306. doi: 10.1186/s12974-026-03964-3 (PMC13555986; doi:10.1186/s12974-026-03964-3)
Supplement: Supplementary file 1 [file 12974_2026_3964_MOESM1_ESM.zip › Fig S3_AS.pdf]

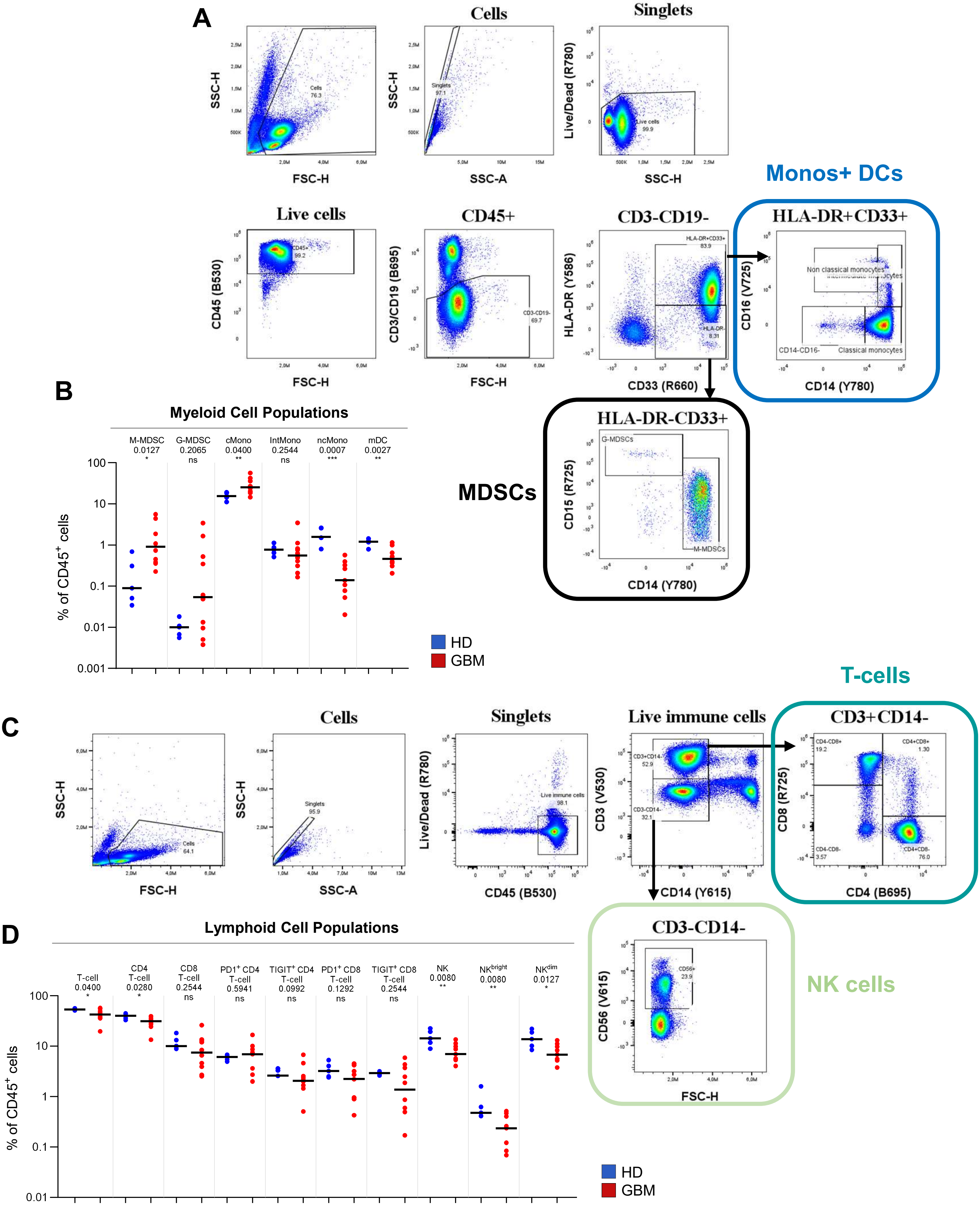
A
Cells
Singlets
SSC-H
FSC-H
SSC-A
Live/Dead (R780)
SSC-H
Cells 76.3
Singlets 97.1
Live cells 99.9
Monos+ DCs
Live cells
CD45+
CD3-CD19-
HLA-DR+CD33+
CD45 (B530)
CD3/CD19 (B695)
HLA-DR (Y586)
CD16 (V725)
FSC-H
FSC-H
CD33 (R660)
CD14 (Y780)
CD45+ 99.2
CD3-CD19- 69.7
HLA-DR+CD33+ 83.9
HLA-DR- 8.31
Non classical monocytes
Intermediate monocytes
CD14-CD16-
Classical monocytes
B
Myeloid Cell Populations
M-MDSC 0.0127 *
G-MDSC 0.2065 ns
cMono 0.0400 **
IntMono 0.2544 ns
ncMono 0.0007 ***
mDC 0.0027 **
% of CD45+ cells
HD
GBM
MDSCs
HLA-DR-CD33+
G-MDSCs
M-MDSCs
CD15 (R725)
CD14 (Y780)
T-cells
C
Cells
Singlets
Live immune cells
CD3+CD14-
SSC-H
SSC-H
Live/Dead (R780)
CD3 (V530)
CD8 (R725)
FSC-H
SSC-A
CD45 (B530)
CD14 (Y615)
CD4 (B695)
Cells 64.1
Singlets 95.9
Live immune cells 98.1
CD3+CD14- 52.9
CD3-CD14- 32.1
CD4-CD8+ 19.2
CD4+CD8+ 1.30
CD4-CD8- 3.57
CD4+CD8- 76.0
D
Lymphoid Cell Populations
T-cell 0.0400 *
CD4 T-cell 0.0280 *
CD8 T-cell 0.2544 ns
PD1+ CD4 T-cell 0.5941 ns
TIGIT+ CD4 T-cell 0.0992 ns
PD1+ CD8 T-cell 0.1292 ns
TIGIT+ CD8 T-cell 0.2544 ns
NK 0.0080 **
NKbright 0.0080 **
NKdim 0.0127 *
% of CD45+ cells
HD
GBM
CD3-CD14-
CD56 (V615)
FSC-H
CD56+ 23.9
NK cells
