# Supplementary material for: Circulating immune profiling reveals impaired monocyte states and trajectories driving immunosuppression in glioblastoma
Source: J Neuroinflammation. 2026 Jul 16;23:306. doi: 10.1186/s12974-026-03964-3 (PMC13555986; doi:10.1186/s12974-026-03964-3)
Supplement: Supplementary file 1 [file 12974_2026_3964_MOESM1_ESM.zip › Fig S4_AS.pdf]

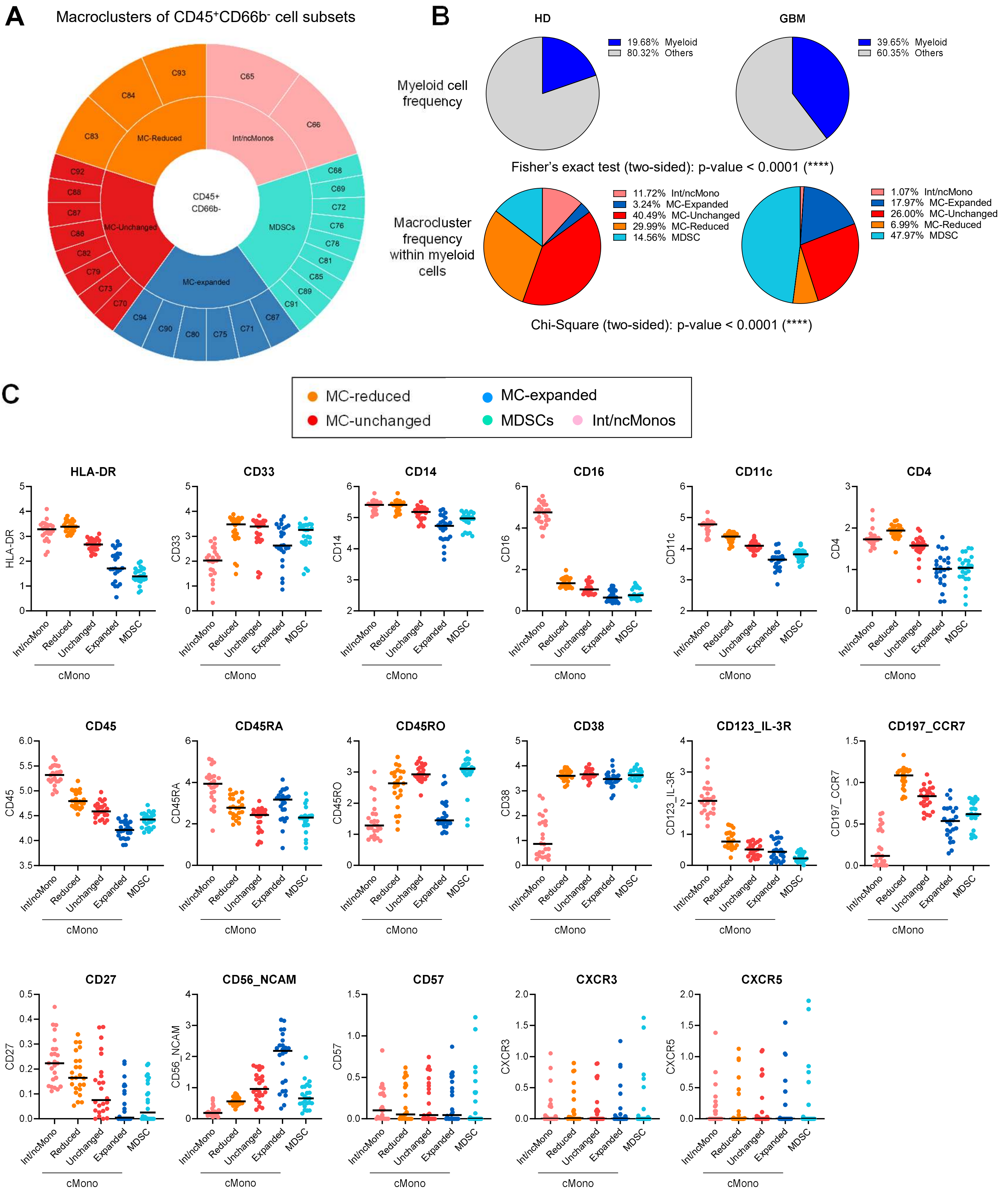

A
Macroclusters of CD45+CD66b- cell subsets
CD45+ CD66b-
MC-Reduced
Int/ncMonos
MDSCs
MC-expanded
MC-Unchanged
C93
C84
C83
C65
C66
C68
C69
C72
C76
C78
C81
C85
C89
C91
C87
C71
C75
C80
C90
C94
C70
C73
C79
C82
C86
C87
C88
C92
B
HD
GBM
Myeloid cell frequency
19.68% Myeloid
80.32% Others
39.65% Myeloid
60.35% Others
Fisher's exact test (two-sided): p-value < 0.0001 (****)
Macrocluster frequency within myeloid cells
11.72% Int/ncMono
3.24% MC-Expanded
40.49% MC-Unchanged
29.99% MC-Reduced
14.56% MDSC
1.07% Int/ncMono
17.97% MC-Expanded
26.00% MC-Unchanged
6.99% MC-Reduced
47.97% MDSC
Chi-Square (two-sided): p-value < 0.0001 (****)
C
MC-reduced
MC-unchanged
MC-expanded
MDSCs
Int/ncMonos
HLA-DR
CD33
CD14
CD16
CD11c
CD4
CD45
CD45RA
CD45RO
CD38
CD123_IL-3R
CD197_CCR7
CD27
CD56_NCAM
CD57
CXCR3
CXCR5
Int/ncMono
Reduced
Unchanged
Expanded
MDSC
cMono
