# Supplementary material for: Circulating immune profiling reveals impaired monocyte states and trajectories driving immunosuppression in glioblastoma
Source: J Neuroinflammation. 2026 Jul 16;23:306. doi: 10.1186/s12974-026-03964-3 (PMC13555986; doi:10.1186/s12974-026-03964-3)
Supplement: Supplementary file 1 [file 12974_2026_3964_MOESM1_ESM.zip › Fig S5_AS.pdf]

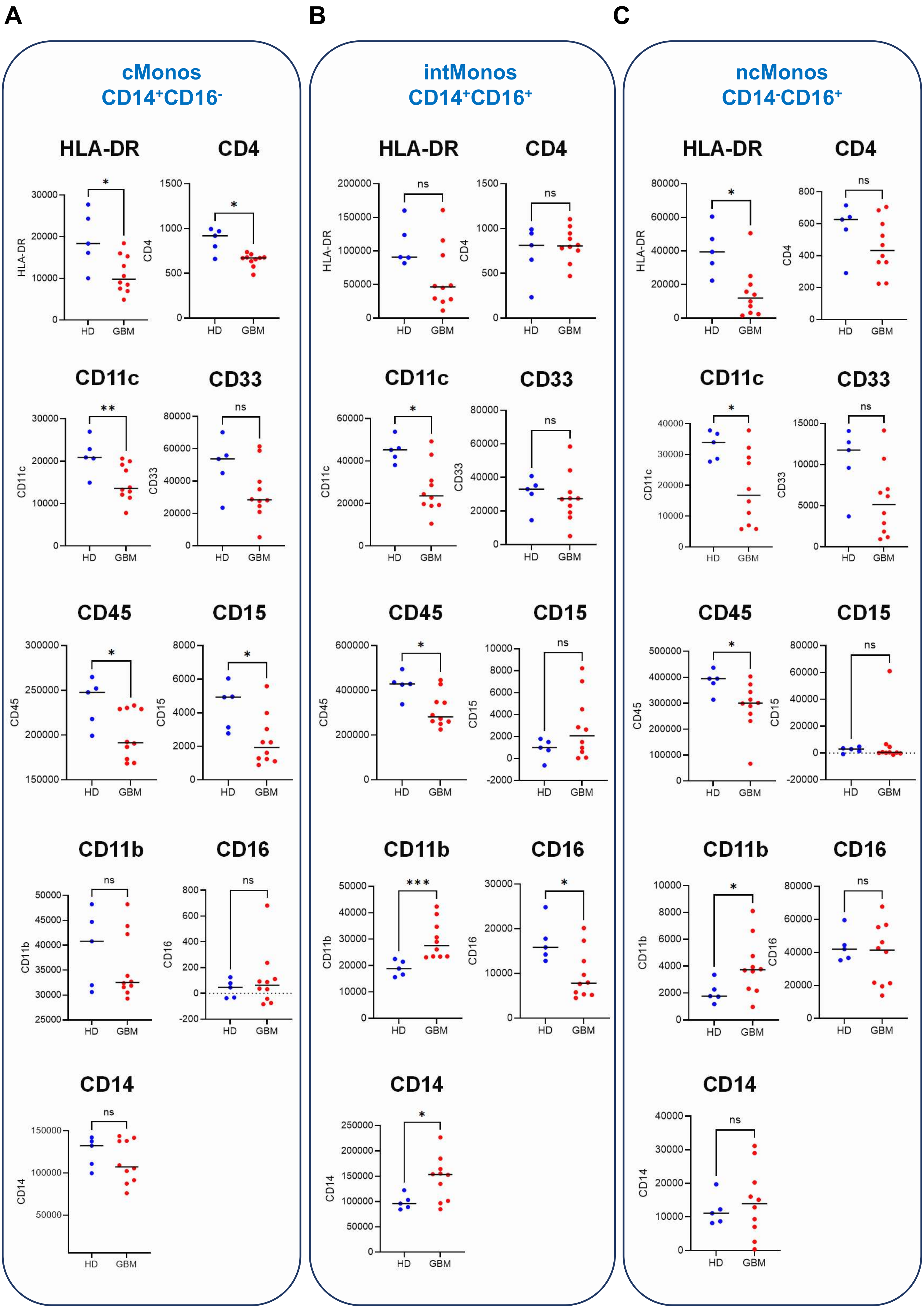
A
B
C
cMonos
CD14+CD16-
intMonos
CD14+CD16+
ncMonos
CD14-CD16+
HLA-DR
CD4
CD11c
CD33
CD45
CD15
CD11b
CD16
CD14
HD
GBM
*
**
***
ns
