# Supplementary material for: Circulating immune profiling reveals impaired monocyte states and trajectories driving immunosuppression in glioblastoma
Source: J Neuroinflammation. 2026 Jul 16;23:306. doi: 10.1186/s12974-026-03964-3 (PMC13555986; doi:10.1186/s12974-026-03964-3)
Supplement: Supplementary file 1 [file 12974_2026_3964_MOESM1_ESM.zip › Fig S6_AS.pdf]

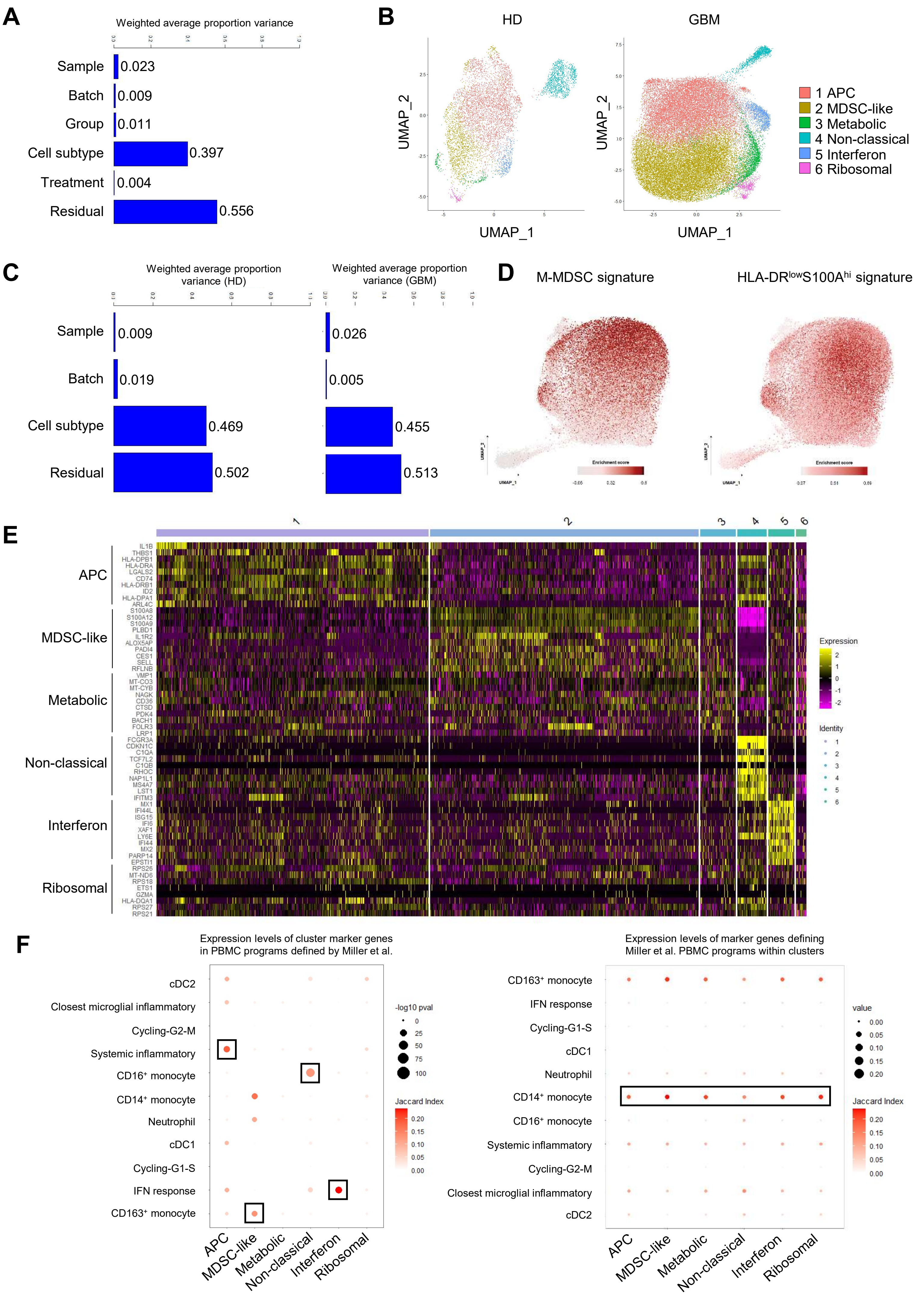

A
Weighted average proportion variance
Sample 0.023
Batch 0.009
Group 0.011
Cell subtype 0.397
Treatment 0.004
Residual 0.556
B
HD
GBM
UMAP_2
UMAP_1
1 APC
2 MDSC-like
3 Metabolic
4 Non-classical
5 Interferon
6 Ribosomal
C
Weighted average proportion variance (HD)
Weighted average proportion variance (GBM)
Sample 0.009 0.026
Batch 0.019 0.005
Cell subtype 0.469 0.455
Residual 0.502 0.513
D
M-MDSC signature
HLA-DRlowS100Ahi signature
Enrichment score
E
APC
MDSC-like
Metabolic
Non-classical
Interferon
Ribosomal
Expression
Identity
F
Expression levels of cluster marker genes in PBMC programs defined by Miller et al.
Expression levels of marker genes defining Miller et al. PBMC programs within clusters
cDC2
Closest microglial inflammatory
Cycling-G2-M
Systemic inflammatory
CD16+ monocyte
CD14+ monocyte
Neutrophil
cDC1
Cycling-G1-S
IFN response
CD163+ monocyte
-log10 pval
Jaccard Index
value
APC
MDSC-like
Metabolic
Non-classical
Interferon
Ribosomal
