# Supplementary material for: Circulating immune profiling reveals impaired monocyte states and trajectories driving immunosuppression in glioblastoma
Source: J Neuroinflammation. 2026 Jul 16;23:306. doi: 10.1186/s12974-026-03964-3 (PMC13555986; doi:10.1186/s12974-026-03964-3)
Supplement: Supplementary file 1 [file 12974_2026_3964_MOESM1_ESM.zip › Fig S7_AS.pdf]

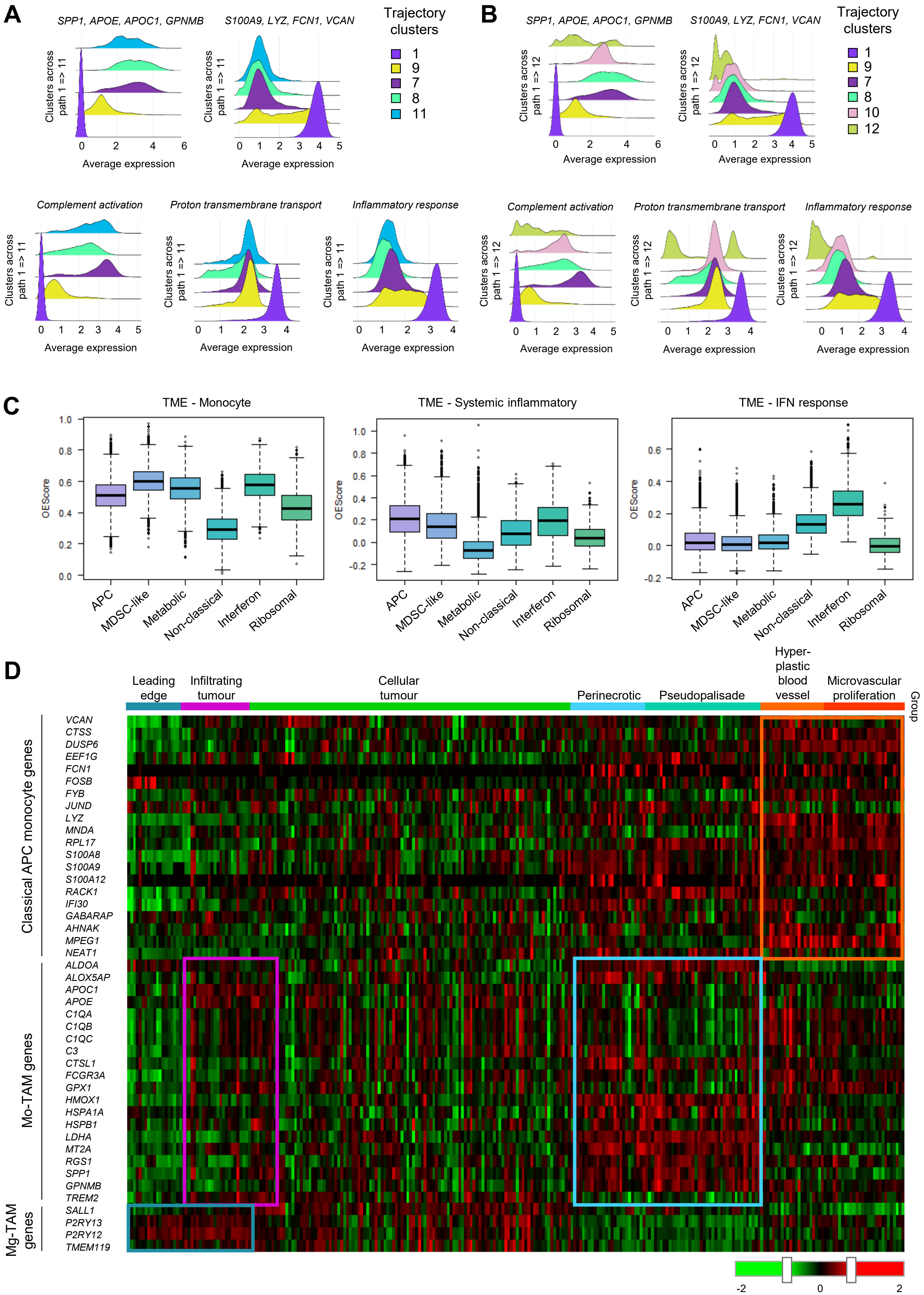

A
SPP1, APOE, APOC1, GPNMB
S100A9, LYZ, FCN1, VCAN
Trajectory clusters
1
9
7
8
11
Clusters across path 1 => 11
Average expression
Complement activation
Proton transmembrane transport
Inflammatory response
B
Trajectory clusters
1
9
7
8
10
12
Clusters across path 1 => 12
C
TME - Monocyte
TME - Systemic inflammatory
TME - IFN response
OEScore
APC
MDSC-like
Metabolic
Non-classical
Interferon
Ribosomal
D
Leading edge
Infiltrating tumour
Cellular tumour
Perinecrotic
Pseudopalisade
Hyper-plastic blood vessel
Microvascular proliferation
Group
Classical APC monocyte genes
VCAN
CTSS
DUSP6
EEF1G
FCN1
FOSB
FYB
JUND
LYZ
MNDA
RPL17
S100A8
S100A9
S100A12
RACK1
IFI30
GABARAP
AHNAK
MPEG1
NEAT1
Mo-TAM genes
ALDOA
ALOX5AP
APOC1
APOE
C1QA
C1QB
C1QC
C3
CTSL1
FCGR3A
GPX1
HMOX1
HSPA1A
HSPB1
LDHA
MT2A
RGS1
SPP1
GPNMB
TREM2
Mg-TAM genes
SALL1
P2RY13
P2RY12
TMEM119
-2
0
2
