# Supplementary material for: Circulating immune profiling reveals impaired monocyte states and trajectories driving immunosuppression in glioblastoma
Source: J Neuroinflammation. 2026 Jul 16;23:306. doi: 10.1186/s12974-026-03964-3 (PMC13555986; doi:10.1186/s12974-026-03964-3)
Supplement: Supplementary file 1 [file 12974_2026_3964_MOESM1_ESM.zip › Fig S8_AS.pdf]

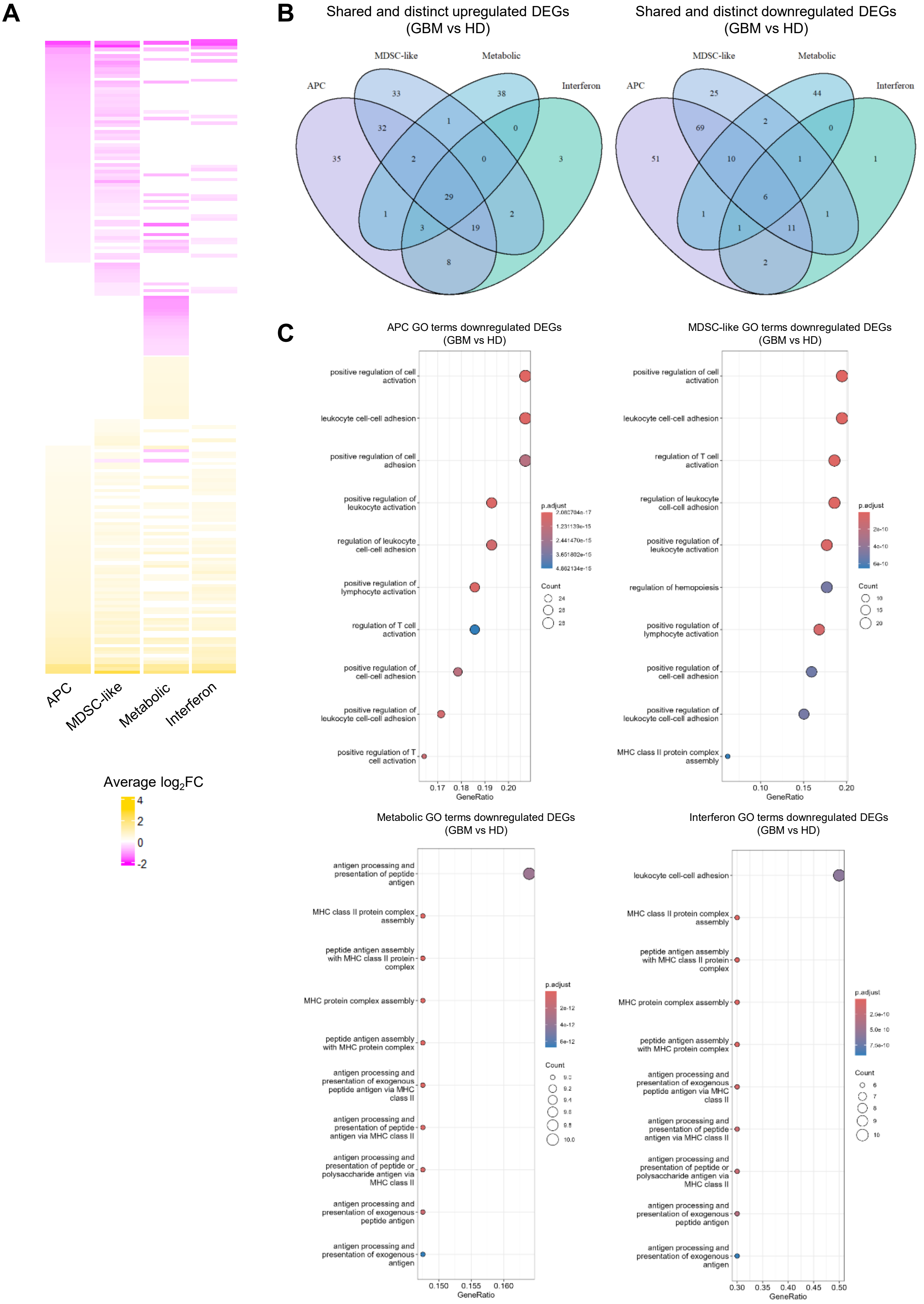

A
APC
MDSC-like
Metabolic
Interferon
Average log2FC
4
2
0
-2
B
Shared and distinct upregulated DEGs (GBM vs HD)
APC
MDSC-like
Metabolic
Interferon
33
38
32
1
0
35
2
0
3
29
1
2
3
19
8
Shared and distinct downregulated DEGs (GBM vs HD)
APC
MDSC-like
Metabolic
Interferon
25
44
69
2
0
51
10
1
1
6
1
1
1
11
2
C
APC GO terms downregulated DEGs (GBM vs HD)
positive regulation of cell activation
leukocyte cell-cell adhesion
positive regulation of cell adhesion
positive regulation of leukocyte activation
regulation of leukocyte cell-cell adhesion
positive regulation of lymphocyte activation
regulation of T cell activation
positive regulation of cell-cell adhesion
positive regulation of leukocyte cell-cell adhesion
positive regulation of T cell activation
0.17
0.18
0.19
0.20
GeneRatio
p.adjust
Count
MDSC-like GO terms downregulated DEGs (GBM vs HD)
positive regulation of cell activation
leukocyte cell-cell adhesion
regulation of T cell activation
regulation of leukocyte cell-cell adhesion
positive regulation of leukocyte activation
regulation of hemopoiesis
positive regulation of lymphocyte activation
positive regulation of cell-cell adhesion
positive regulation of leukocyte cell-cell adhesion
MHC class II protein complex assembly
0.10
0.15
0.20
GeneRatio
p.adjust
Count
Metabolic GO terms downregulated DEGs (GBM vs HD)
antigen processing and presentation of peptide antigen
MHC class II protein complex assembly
peptide antigen assembly with MHC class II protein complex
MHC protein complex assembly
peptide antigen assembly with MHC protein complex
antigen processing and presentation of exogenous peptide antigen via MHC class II
antigen processing and presentation of peptide antigen via MHC class II
antigen processing and presentation of peptide or polysaccharide antigen via MHC class II
antigen processing and presentation of exogenous peptide antigen
antigen processing and presentation of exogenous antigen
0.150
0.155
0.160
GeneRatio
p.adjust
Count
Interferon GO terms downregulated DEGs (GBM vs HD)
leukocyte cell-cell adhesion
MHC class II protein complex assembly
peptide antigen assembly with MHC class II protein complex
MHC protein complex assembly
peptide antigen assembly with MHC protein complex
antigen processing and presentation of exogenous peptide antigen via MHC class II
antigen processing and presentation of peptide antigen via MHC class II
antigen processing and presentation of peptide or polysaccharide antigen via MHC class II
antigen processing and presentation of exogenous peptide antigen
antigen processing and presentation of exogenous antigen
0.30
0.35
0.40
0.45
0.50
GeneRatio
p.adjust
Count
