# Supplementary material for: Circulating immune profiling reveals impaired monocyte states and trajectories driving immunosuppression in glioblastoma
Source: J Neuroinflammation. 2026 Jul 16;23:306. doi: 10.1186/s12974-026-03964-3 (PMC13555986; doi:10.1186/s12974-026-03964-3)
Supplement: Supplementary file 1 [file 12974_2026_3964_MOESM1_ESM.zip › Fig S9_AS.pdf]

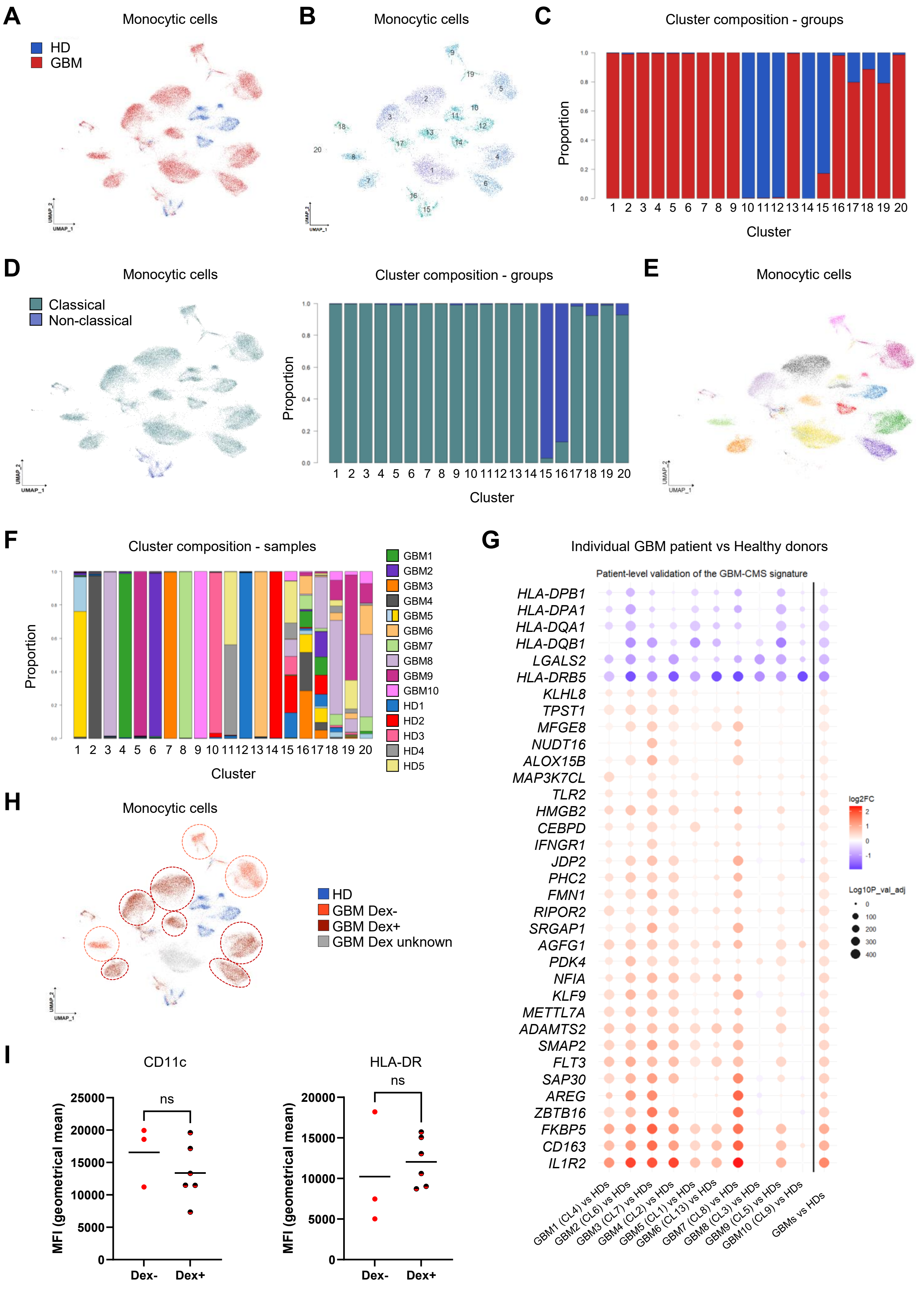
A
Monocytic cells
HD
GBM
B
Monocytic cells
C
Cluster composition - groups
Proportion
Cluster
D
Monocytic cells
Classical
Non-classical
Cluster composition - groups
Proportion
Cluster
E
Monocytic cells
F
Cluster composition - samples
Proportion
Cluster
GBM1
GBM2
GBM3
GBM4
GBM5
GBM6
GBM7
GBM8
GBM9
GBM10
HD1
HD2
HD3
HD4
HD5
G
Individual GBM patient vs Healthy donors
Patient-level validation of the GBM-CMS signature
HLA-DPB1
HLA-DPA1
HLA-DQA1
HLA-DQB1
LGALS2
HLA-DRB5
KLHL8
TPST1
MFGE8
NUDT16
ALOX15B
MAP3K7CL
TLR2
HMGB2
CEBPD
IFNGR1
JDP2
PHC2
FMN1
RIPOR2
SRGAP1
AGFG1
PDK4
NFIA
KLF9
METTL7A
ADAMTS2
SMAP2
FLT3
SAP30
AREG
ZBTB16
FKBP5
CD163
IL1R2
log2FC
Log10P_val_adj
GBM1 (CL4) vs HDs
GBM2 (CL6) vs HDs
GBM3 (CL7) vs HDs
GBM4 (CL2) vs HDs
GBM5 (CL1) vs HDs
GBM6 (CL13) vs HDs
GBM7 (CL8) vs HDs
GBM8 (CL3) vs HDs
GBM9 (CL5) vs HDs
GBM10 (CL9) vs HDs
GBMs vs HDs
H
Monocytic cells
HD
GBM Dex-
GBM Dex+
GBM Dex unknown
I
CD11c
ns
MFI (geometrical mean)
Dex-
Dex+
HLA-DR
ns
MFI (geometrical mean)
Dex-
Dex+
